# Adaptation of a pediatric hospital to the recommendations of the Brazilian Dietary Guidelines, Niterói, 2023

**DOI:** 10.1590/S2237-96222025v34e20240532.en

**Published:** 2025-08-08

**Authors:** Amine Farias Costa, Letícia Ferreira Tavares, Paulo Cesar Pereira de Castro, Raphael Barreto da Conceição Barbosa, Olivia Souza Honório, Ana Carolina Rocha Oliveira, Letícia de Oliveira Cardoso

**Affiliations:** 1 Instituto Nacional de Câncer, Hospital do Câncer II, Rio de Janeiro, RJ, Brazil; 2 Universidade Federal do Rio de Janeiro, Instituto de Nutrição Josué de Castro, Departamento de Gastronomia, Rio de Janeiro, RJ, Brazil; 3 Universidade Federal do Rio de Janeiro, Instituto de Nutrição Josué de Castro, Departamento de Nutrição Social Aplicada, Rio de Janeiro, RJ, Brazil; 4 Instituto Desiderata, Rio de Janeiro, RJ, Brazil; 5 Fundação Oswaldo Cruz, Escola Nacional de Saúde Pública Sergio Arouca, Rio de Janeiro, RJ, Brazil

**Keywords:** Environment, Food, Hospital, Pediatrics, Observational Study, Medio ambiente, Alimento, Hospital, Pediatría, Estudio Observacional

## Abstract

**Objective:**

To evaluate the adequacy of a pediatric hospital in a large city in the metropolitan region of Rio de Janeiro to the recommendations of the Brazilian Dietary Guidelines regarding the service provision contract, dietary prescription and aspects of the food environment.

**Methods:**

Observational study evaluating four dimensions of the hospital food environment and its surroundings: current legislation and food service provision contract; prescribed diets, the menu and technical sheets of preparations; infrastructure of the Food and Nutrition Unit; and the sale and advertising of food in the internal and external food environment. Technical visits were conducted to evaluate documents, assess menus and conduct direct observation, using previously validated instruments.

**Results:**

The service provision contract was fully compliant for the infant formulas and enteral diets, and the menu was partially compliant (81%). Most of the preparations analyzed were classified as high quality or intermediate quality (77%). Considering the infrastructure of the Food and Nutrition Unit, 90%, 89% and 71% of the responses were positive for essential, recommended and necessary items, respectively. The hospital’s food environment stood out for offering in nature and minimally processed foods. However, there was a wide variety of ultra-processed foods available in self-service machines and from street vendors.

**Conclusion:**

Despite a satisfactory food environment, the presence and sale of ultra-processed foods was identified in the hospital and its surroundings.

## Resumen

**Objetivo:**

analizar la adecuación de un hospital pediátrico, de una gran ciudad de la región metropolitana de Río de Janeiro, a las recomendaciones de las guías alimentarias cuanto al contrato de prestación de servicios, prescripción dietética y aspectos del ambiente alimentario.

**Métodos:**

Estudio observacional que analizó cuatro dimensiones del ambiente alimentario hospitalario y su entorno: legislación vigente y contrato de prestación del servicio de alimentación; dietas prescritas, menú elaborado y fichas técnicas de preparaciones; infraestructura de la Unidad de Alimentación y Nutrición; y la venta y publicidad de alimentos en el ámbito alimentario interno y externo. Se realizaron visitas técnicas para evaluar documentos, analizar menús y realizar observación directa, utilizando instrumentos previamente validados.

**Resultados:**

El contrato de prestación de servicios cumplió totalmente con lo establecido para fórmulas infantiles y dietas enterales, y parcialmente (81%) para el menú. La mayoría de las preparaciones analizadas fueron clasificadas como de calidad alta o calidad intermedia (77%). En la infraestructura de la Unidad de Alimentación y Nutrición, el 90%, 89% y 71% de las respuestas fueron positivas para los artículos esenciales, recomendados y necesarios, respectivamente. El ambiente alimentario del hospital se destacó por ofrecer alimentos naturales y mínimamente procesados. Sin embargo, había una amplia variedad de alimentos ultraprocesados disponibles en máquinas de autoservicio y en vendedores ambulantes.

**Conclusión:**

A pesar de un ambiente alimentario satisfactorio, se identificó la presencia y venta de alimentos ultraprocesados en el hospital y su entorno.

**Ethical aspects**

This research used public domain data and anonymized databases.:

## Introduction

Food plays a fundamental role throughout all stages of life, especially in the early years, and directly influences the process of growth and development, the formation of new habits, the maintenance of health and the formation of human capital in a society. Inadequate nutrition, and consequently malnutrition, in its different expressions, from malnutrition to obesity, is one of the main public health problems today (1-3). 

In recent decades, Brazil has invested in implementing public policies to promote, protect and support healthy eating for children and young people. However, despite the advances, the consumption of ultra-processed foods continues to grow in this age group. Among oooher reasons, this increase is associated with early exposure to these foods, to the detriment of the consumption of in nature or minimally processed foods (1,4). The consumption of ultra-processed foods has been associated with several health outcomes in this population, such as overweight, obesity (4.5), and cardiometabolic comorbidities (6.7).

The food environment is characterized by a set of physical, economic, political and sociocultural conditions that directly influence food choices and the nutritional status of individuals, including children and adolescents (8,9).

Hospitals and their respective food service locations are considered organizational food environments, and they are the target of public policies and national and international recommendations for the promotion of adequate and healthy eating habits (10,11). 

The current Brazilian National Food and Nutrition Policy has among its guidelines the creation and promotion of health-friendly food environments in which individuals and communities can make healthy choices and foresees the implementation of actions that protect individuals from environments that encourage unhealthy choices and practices, through regulatory measures (12,13). 

Considering that the hospital, as an institution, acts as a place for recovering the health of individuals and encouraging healthy lifestyle habits, and that it is essential that its practices are in line with the guidelines recommended by SUS (Brazilian Unified Health System), the objective of this study was to analyze the adequacy of a pediatric hospital, in a large city in the metropolitan region of Rio de Janeiro, to the recommendations of the Dietary Guidelines regaridng the service provision contract, dietary prescription and aspects of the food environment. 

## Methods

### Design

This is an observational, cross-sectional, hospital-based study conducted between January and May of 2023.

### **Study context and size**.

The study was conducted in a public pediatric hospital located in the municipality of Niterói, metropolitan region of Rio de Janeiro, State of Rio de Janeiro, Brazil. The hospital evaluated is a reference in pediatric care in the region, performing around 6 thousand emergency care, 2 thousand specialized consultations and an average of 180 hospitalizations each month (14). 

Compliance with the recommendations of the Dietary Guidelines was assessed through local technical visits, divided into four modules: 1. assessment of current legislation and the contract for the provision of food and nutrition services implemented in the hospital; 2. assessment of the prescribed diets, the menu, the technical sheets and their comparison with the guidelines of the contract; 3. assessment of the infrastructure of the Food and Nutrition Unit and the cafeteria for preparing and distributing meals; 4. evaluation of the internal and external food environment with regard to the sale and advertising of food. 

### Variables

### **Module 1**:

An analysis was carried out of the food and nutrition service provision contract implemented in the hospital and of the current legislation on food in pediatric hospitals, aiming to: (a) evaluate the guidelines for the preparation of children’s menus, as well as their structure, composition, frequency of food offering, characteristics of hospital diets and permitted and prohibited items; (b) evaluate the preparation, composition (essential and optional ingredients) and labeling of infant formulas; (c) evaluate the prescription, preparation, transportation, administration, clinical and laboratory control, labeling and packaging of enteral diets, and other stages of the process involving enteral nutritional therapy.

Table 1 presents the items analyzed in the evaluation of menus, infant formulas and enteral diets. 

**Table 1:** List of items analyzed in the evaluation of distributed menus, infant formulas and enteral diets to the public treated at a public pediatric hospital in Niterói

Item (absolute quantity)	ItemdDescription
**Menus** (14)	
General guidelines (7)	- Preparation of monthly menus. - Technical data sheets for food preparations.^a^ - Planning for different menus on festive dates. - Fixing the menu in a visible place. - Menu changes with at least 24-hour notice. - Provision of sauces and seasonings to increase acceptability. - Planning for special foods for specific pathologies.
Structure (1)	- Basic composition of meals for children.
Composition and frequency (4)	- Level of food processing.^a^ - Food groups. - Frequency of food groups offered. - Nutritional composition.
Characteristics of hospital diets (2)	- Basic diets. - Restrictive diets.
Food items (2)	- Allowed. - Forbidden.^a^
**Infant formulas** (16)	
Definition (4)	- Infant formulas for infants. - Follow-up formulas for infants and toddlers. - Infant formulas intended for specific dietary needs. - Follow-on infant formulas for infants and toddlers designed for specific dietary needs.
Preparation (2)	- Label instructions. - Quantity according to the child’s weight and age.
Essential composition (3)	- Macronutrient recommendations. - Micronutrient recommendations. - Gluten free.
Optional ingredients (1)	- Specific rules for the use of taurine, nucleotides, docosahexaenoic acid, fructooligosaccharides, galactooligosaccharides and probiotics.
Labeling (5)	- Nutritional information for 100g or 100mL. - Optional ingredients with nutritional information clearly visible. - Absence of daily value percentage. - Presence of mandatory information on infant formulas intended for dietary therapy needs - Presence of warning phrases on infant formulas.
Dairy compound (1)	- Presence of warnings.
**Enteral Diets** (45)	
Definition (3)	- Composition. - Isolated or combined nutrients. - Open or closed system.
General conditions (8)	- Indication. - Medical prescription. - Dietary prescription, preparation. - Conservation and storage. - Transportation. - Administration. - Clinical laboratory control. - Final evaluation.
Indication and prescription (3)	- Medical indication. - Diet therapy prescription. - Prior nutritional assessment.
Preparation and conservation (7)	- Assessment of dietary prescription. - Manipulation. - Quality control. - Conservation. - Certificate of analysis for raw materials and containers. - Labeling. - Sample reservation.
Transport and administration (3)	- Quality maintenance. - Transportation. - Administration.
Clinical and laboratory control (1)	- Clinical and laboratory control of the patient.
Nutritionist’s role (1)	- List of private assignments.
Good preparation practices (3)	- Diet preparation. - Acquisition of raw materials and packaging materials. - Acquisition of industrialized diets.
Specifications for formulas (4)	- Formula for enteral nutrition. - Standard formula for enteral nutrition. - Modified formula for enteral nutrition. - Module for enteral nutrition.
Labeling and packaging (12)	- Complete patient identification - Qualitative and quantitative composition of all components. - Total volume. - Rate of administration. - Access route. - Date and time of manipulation. - Expiration date. - Control sequence number. - Temperature conditions for conservation. - Name and Professional Board number of the person technically responsible for the process. - Absence of misleading information. - Specific labeling requirements for age-specific modified formulas.

^a^Not compliant.

### **Module 2**:

A documentary analysis was conducted to assess all food products and processed foods purchased, in addition to culinary preparations cooked on site, and distributed to the public served by the hospital.

### **Module 3**:

The following variables related to the infrastructure of the Food and Nutrition Unit and cafeteria were evaluated: documentation and records; standardized operating procedures; physical facilities; fire-fighting system; walls, floors, doors and windows; electrical installations, lighting and ventilation; changing rooms and toilets; sink for hand hygiene; garbage, grease traps and sewage; control of vectors and urban pests; water supply; equipment and utensils; hygiene of the physical spaces, facilities and utensils; hygiene of handlers; production - acquisition and receipt of raw materials; production - storage; production - food handling; production - distribution and consumption of food; quality control and transportation of food; worker and user satisfaction.

### **Module 4**:

The assessment of the hospital’s food environment included an analysis of three types of services: cafeteria, self-service machines and informal street vendors present in the immediate surroundings of the location. 

Characteristics of the cafeteria, such as physical infrastructure and equipment, type of drinks, food and preparations offered, prices and promotions, advertising, consumer information, and opening hours, were evaluated. Considering self-service machines, the supply, availability, quality, advertising and price of available food and drinks were evaluated. Street vendors around the hospital were assessed in terms of its structure, types and variety of food sold and advertising used.

### Data Sources and Measurement

### **Module 1**:

For the evaluation of the menu, infant formulas and enteral diets, the Dietary Guidelines for the Brazilian Population (2) and the Dietary Guidelines for Children Under 2 Years Old (1), referred to hereinafter as “national dietary guidelines”, were used, in addition to programs and standards of the Brazilian government and national regulatory agencies legislation (16-24). 

### **Module 2**:

The menus and preparations of all meals, the composition of the baby bottles prepared, the requests for food products orders, the labels of the processed foods used in food preparations or that were distributed to pediatric patients at the hospital and the meal planning maps of the Food and Nutrition Unit and of the prescribing nutritionists were analyzed. 

Three random and non-consecutive days (two weekdays and one weekend day) were analyzed for the lunch and dinner menu, for the two available consistencies (soft and pasty). The hospital’s Food and Nutrition Unit did not have a menu plan for small meals (breakfast, afternoon tea, snack and supper), therefore, the food offered to patients on the day of the technical visit to the hospital were analyzed. 

### **Module 3**:

The data were collected through observation and analysis of the Food and Nutrition Unit and cafeteria guided by an instrument constructed and validated based on the dimensions of structure, processes and results, informed by the general legislation on food, scientific literature, and instruments already used by some Health Surveillance services (25).

The instrument used in data collection has a list of 227 items, of which 131 are considered essential (those that could critically influence the quality and safety of meals and the safety of workers during the production process), 89 necessary (when they can influence the quality and safety of meals and the safety of workers during the process to a less critical degree) and 7 recommended (when they can influence the quality and safety of workers’ meals to a non-critical degree during the process).

### **Module 4**:

The cafeteria food environment was evaluated using an instrument developed specifically for the hospital area - “Hospital Food Environment Assessment Instrument,” which in a previous analysis showed good reliability in the context in which it was used (26). The instrument, in the form of a checklist, contains 8 blocks for evaluating the food environment, and for this analysis, only block D, intended for evaluating the hospital cafeteria, was used. The block contains 41 items that were checked based on observation of the environment evaluated. 

To evaluate the self-service machines, a validated checklist-type instrument was used, which contains 3 blocks: identification of the evaluator, the establishment, the study location and the data collection; information about the self-service machine, such as location, food and/or drinks available, specific brand, payment methods and presence of advertising; and information about the food items available (25 options among food and drinks). (27-28) 

Street vendors in the area surrounding the hospital were assessed using an instrument developed to assess street trading in the area surrounding Brazilian private schools (29). The instrument, in the form of a checklist, contains 129 items, divided into 3 sections, which include questions about general data on street food trade; sociodemographic data of street vendors; structure of the trade (cart, stall, and others); food and beverages sold, with details on the variety offered, size and price of portions, and sales strategies; advertising used for the food sold and whether the vendor has ever been prevented or prohibited from operating on the premises by the government, police or the population.

### Statistical Methods

### **Module 1**:

The information collected in the documents used in the hospital was assessed for compliance (compliant or non-compliant) with the standards and legislation used as a reference for these analyses, and the results were presented as absolute and relative frequencies.

### **Module 2**:

The Score for Qualitative Assessment of Preparation (30) was used to classify each food item according to its degree of industrial processing and following the recommendations of national dietary guidelines (1.4). The evaluation of the preparations was based on a questionnaire consisting of ten questions that evaluated and scored, in addition to the industrial processing of the ingredient, the use of ingredients in nature as the main ingredientes and in greater quantity; the presence or absence of animal protein, or fish with less fat; the use of cereals and seeds in their whole form; the presence of sugar, brown sugar, honey or molasses; and the presence of fried foods by immersion in oil. To apply this tool, we used the Microsoft Excel spreadsheet provided by the authors of the method, which organized the preparations by group (salad, main course, side dish, garnish and dessert). The ingredients of each preparation and the information on the labels of each product used in the preparation were included in this spreadsheet. The score and classification of the preparations according to their score was automatically filled in after answering each question. Each food preparation was classified according to the Score for Qualitative Assessment of Preparation, namely: ≥11, high quality preparation; 6 to 10, intermediate quality; 0 to 5, low quality; ≤-1, very low quality (30).

### **Module 3**:

The percentage of positive responses was used for all items evaluated in the hospital. The establishment is classified as good condition when it has 91% or more positive responses for essential items and 71% or more for necessary items and is classified as in satisfactory condition when it has 70% to 90% positive responses for essential items and 50% to 70% for necessary items (25).

### **Module 4**:

The indicator used was the ratio between the availability of ultra-processed foods and culinary preparations containing these foods and in nature foods, minimally processed or processed and culinary preparations based on these foods to assess the availability of food from self-service machines and street vendors (31).

## Results

Considering the reference documents and legislation assessed in module 1, the food and nutrition service provision contract implemented in the hospital was fully compliant (Table 1) regarding infant formulas and enteral diets. Regarding the menu, 16 items were evaluated and 3 (19%) were classified as non-compliant - absence of technical sheets for preparations, use of ultra-processed foods, and use of prohibited items (salt and sugar). Although the contract emphasizes that the patient’s diet must follow nutritional recommendations, there is no reference to the national dietary guidelines.

Most of the preparations analyzed were classified as high quality or intermediate quality (77%). All savory preparations, distributed at lunch and dinner, were classified as high quality (42%) according to the score. Preparations classified as intermediate quality (35%), low quality (12%) and very low quality (10%) were those that did not contain in nature food in their composition, and those that had added sugars, artificial sweeteners or ultra-processed foods (Table 2).

**Table 2:** Qualitative evaluation score of food products, processed foods and culinary preparations prepared and distributed to the public served in a pediatric hospital of the Brazilian public health services (SUS) in Niterói

Preparation	Score
High quality	
Potato dumplings with chicken	13
Assorted fruits (whole, cream or puree)	13
Assorted sauteed vegetables	13
Orange juice	13
Mixed vegetable puree	13
White rice	12
Red meat (steak or ground)	12
Mulatto beans	12
Chicken (grilled or shredded breast fillet)	12
Noodles	12
Bottle: water, whole cow’s milk powder (13%)	12
Banana jam	11
Bottle: whole cow’s milk, oats (5%), sugar (5%)	(11)
Oatmeal porridge (whole cow’s milk, oats and sugar)	11
Fruit juice (frozen fruit pulp, water and sugar)	11
Vegetable omelette	12
Grilled or shredded fish	12
Intermediate quality	
Pudding	10
Whole milk with coffee and sugar	10
Bottle: whole cow’s milk, cornstarch (5%), sugar (5%)	10
Cornstarch porridge (whole cow’s milk, cornstarch and sugar)	10
Fruit smoothie (whole cow’s milk, fruit and sugar)	10
Fruit juice (frozen fruit pulp, water and artificial sweetener)	7
Whole milk with coffee and artificial sweetener	6
Whole milk with regular or diet chocolate	6
Bottle: whole cow’s milk, milk flour^a^ (5%)	6
Bottle: whole cow’s milk (75%), water, multigrain flour^a^ (5%), maltodextrin (5%)	6
Oatmeal porridge (whole cow’s milk, oats and artificial sweetener)	6
Milk flour porridge (whole cow’s milk and milk flour)^a^	6
Cornstarch porridge (whole cow’s milk, cornstarch and sweetener)	6
Fruit smoothie (whole cow’s milk, fruit and artificial sweetener)	6
Low quality	
Bottle: whole cow’s milk, multigrain flour^a^ (5%), sugar (5%)	4
Bottle: whole cow’s milk, rice flour^a^ (5%), sugar (5%)	4
Bottle: whole cow’s milk, multigrain flour^a^ (5%), sugar (5%)	4
Bottle: whole cow’s milk, rice flour^a^ (5%), sugar (5%)	4
French bread with Minas cheese	1
Very low quality	
French bread with margarine	-5
French bread with cheese (“prato” cheese)	-5
Salty or sweet biscuit	-6
“Minissaia” pudding (pudding powder and gelatin powder)^a^	-7

^a^Ultra-processed powdered products.

Regarding module 2, the preparations did not have technical sheets, therefore, the quantities added of each ingredient varied according to the employee who prepared it. However, the quantity of ingredients is not part of the calculation of the Score for Qualitative Preparation Assessment, only information on the presence or absence of a certain ingredient.

Of the 227 questions evaluated in module 3, the percentage of positive responses for the essential, recommended and necessary items was 90%, 89% and 71%, respectively, thus showing satisfactory conditions in meeting the essential items, and good conditions in meeting the necessary items. 

The nonconformities found are concentrated in the lack of temperature records, equipment calibration and microbiological analyses; presence of vectors; and inadequacies in physical areas (Table 3).

**Table 3:** Assessment of the infrastructure of the Food and Nutrition Unit and hospital cafeteria of a pediatric hospital in the public health network (SUS) of Niterói: items with negative responses and corresponding classification.

Item classification^a^ (relative frequency)	Items with negative response
Essential (5%)	- No urban pests in facilities. - Heated food carts for food distribution maintain a temperature of at least 65ºC. - The temperatures of perishable raw materials upon receipt were checked and recorded. - Storage allows ventilation, cleaning and, when necessary, sanitation of the location. - Effectiveness of heat treatment assessed by checking the temperature and time used and, when applicable, changes in texture and color in the central part of the food. - Reheated food reaches 74ºC inside or is subjected to a time and temperature range that guarantees its safety. - If the analysis results are unsatisfactory, what measures should be taken?
Necessary (10%)	- Has an architectural project approved by the Health Surveillance Agency. - Has equipment maintenance and calibration program. - Records of equipment maintenance and calibration. - Standard Operating Procedures for equipment calibration. - Changing rooms and toilets comply with general facilities requirements. - Changing rooms equipped with lockers that were not in good condition. - Thermal gloves are used to avoid direct contact with food. - When stored at room temperature, the physical capacity of the tanks is sufficient. - Elevators for meal transport (when applicable) are exclusive for this use.
Recommended (29%)	- The establishment measures and evaluates employee satisfaction. - There are incentives for professional training offered by the establishment to employees.

^a^ We evaluated 227 items between January and May 2023, of which: 131 items were classified as essential, 89 necessary and 7 recommended. Essential items can have a critical impact on the quality and safety of meals and the safety of workers during the production process; necessary items can have a less critical impact on the quality and safety of meals and the safety of workers during the process; and recommended items can have a non-critical impact on the quality and safety of workers’ meals during the process.

A cafeteria and two self-service machines were found in module 4 assessments. In the surrounding area, there were two street vendors selling food and drinks.

The cafeteria has air conditioning and furniture for dining in. The daily menu is available to users and consists of four meals, with vegetables, cereals and legumes offered in the main meals, and coffee and bread in the small meals. No in nature fruit was identified on the menu evaluated. 

However, self-service machines are installed in the main distribution points for food and drinks with free access to everyone in the hospital, with the predominant supply of ultra-processed foods, and even with advertising incentives for their consumption. Among the ultra-processed foods available for purchase, ready-to-eat ultra-processed beverages, chocolates, candies, and other treats, stuffed biscuits and packaged snacks were found. The same profile of food availability was found in street trading. 

The results of the indicator ratio between the availability of ultra-processed foods and culinary preparations containing these foods and foods in nature, minimally processed or processed and culinary preparations based on these foods show that there is a predominance of availability of ultra-processed foods in self-service machines and street vendors. Street vendors offer 1.3 to 3.5 times more ultra-processed foods and culinary preparations containing these foods than in nature, minimally processed or processed and culinary preparations based on these foods. A self-service machine offers 10 ultra-processed foods and no in nature, minimally processed food or processed and culinary preparations based on these foods and the other provides 50% more ultra-processed foods. 

## Discussion

This study presented data on the evaluation of the food environment in a pediatric hospital located in the metropolitan region of Rio de Janeiro, Brazil. The results show the acquisition, distribution, advertising and sale of ultra-processed foods to children and young people served at the location, as well as to their guardians and workers at the location. It is also worth noting that the hospital’s service provision contract advises following nutritional recommendations but does not reference national dietary guidelines (1,2).

The analysis of the guidelines of the current contract and the management instruments used showed that they are mostly in line with national legislation and documents about food and nutrition. However, the supply and distribution of ultra-processed foods to users is visible, and when observing the analysis of the food environment, the advertising and sale of ultra-processed foods inside and around the hospital are also noted. Therefore, both the supply and the food made available in the environment go against the recommendations of dietary guidelines that advocate the supply of food in nature and minimally processed and advise avoiding ultra-processed foods. 

The consumption of ultra-processed foods is related to the incidence of obesity and chronic non-communicable diseases in adults and children and young people (2). Thus, considering that the purpose of the hospital environment is, among others, the promotion, prevention, recovery and protection of health, the offer, sale and advertising of ultra-processed foods in this environment, even with a set of evidence that highlights their harm to health, distance the place from the intended objectives.

A narrative review, based on studies conducted in the USA and the United Kingdom, pointed to a diversity of foods available in stores inside hospitals (restaurants, cafeterias and self-service machines), but with a low availability of healthy foods. The study also points out that regulatory measures have a positive effect both on increasing the acquisition of healthy foods and on reducing unhealthy foods (32).

In Brazil, a study that aimed to characterize the food environment of the public hospital network, managed by the city government of Rio de Janeiro (n=24), concluded that the food environment of the hospitals studied does not promote healthy eating habits. A high availability of ultra-processed foods was found in the hospital environment, and advertising of these foods is frequent, encouraging the consumption of unhealthy foods (33). The results are in line with those presented here when we observe the foods sold inside the hospital.

The Informas initiative (International Network for Food and Obesity/non-communicable disease Research, Monitoring and Action Support) argues that environments organized and managed by public authorities should be governed by food policies that regulate what should or should not be offered. Also highlighted is the nutritional quality of food made available and/or sold in public spaces, which should be in line with the nutritional guidelines, standards or other guidelines established by the country (34).

It is also worth noting that measures such as the prohibition and restriction of the supply, marketing and advertising of ultra-processed foods have been recommended by different international organizations aiming at containing obesity, especially among children. In recent years, international governance organizations have recommended the protection of environments where children and adolescents are present, and in the context of food environments they highlight the need to build healthy food environments, especially in organizational environments (35-37). 

 In Brazil, some actions to regulate the availability, access, distribution, advertising and publicity of ultra-processed foods, based on scientific evidence and national dietary guidelines (1,2), have been proposed in recent years and could be adopted in order to establish consonance between what is recommended by the Ministry of Health and what is offered in health establishments that are part of the SUS. The Ministry of Health established guidelines for promoting adequate, healthy and sustainable nutrition, to be adopted in actions to promote health and quality of life at work and in environments that sell, produce or offer food within the scope of the Ministry (38), with no regulation as to what should be made available in institutions served by SUS. For children and young people, the National School Feeding Program provides for the prohibition of ultra-processed foods and the addition of sugars and artificial sweeteners in culinary preparations and drinks for children up to three years of age (39) in shcool meals, thus demonstrating the viability and importance of regulatory measures within public network systems. 

To date, there are no studies that evaluate the food environment in pediatric hospitals. The unprecedented nature of the subject requires continuous discussion and the need for cooperation between different actors. In this sense, the results of the present study mobilized the researchers involved in the research team and a panel of judges composed of experts from different areas, which go beyond the health field and the governmental sphere, in the construction of a Technical Note, based on national dietary guidelines, which aims to guide the regulation of the hospital food environment (40).

The present study also has limitations. This is a sectional study in a hospital unit, which is susceptible to observation biases. It is also important to highlight that the assessment of the food environment in the surroundings may undergo changes, given that we analyzed informal street trading on the sidewalk of the health establishment, and its presence is susceptible to factors such as weather conditions and time of day. However, to minimize the risks of these biases, validated and standardized instruments as well as research protocols were used, and field researchers were trained. Furthermore, the food environment collection took place on a day without rain, during business hours. 

The evaluation conducted demonstrated that the hospital’s food environment was generally satisfactory, in terms of compliance with legislation, infrastructure, quality of services provided, and food supplied, however, with the offer, sale and advertising of ultra-processed foods inside and around the site. We expect that this study can contribute to supporting the improvement of regulatory measures in institutional food environments, aiming at access to good quality, healthy and sustainable food for all users of these places.

## Data Availability

This study does not have a database. Additional information may be available on request from the authors.
