# Supplementary material for: Adaptation of a pediatric hospital to the recommendations of the Brazilian Dietary Guidelines, Niterói, 2023
Source: Epidemiol Serv Saude. 2025 Aug 8;34:e20240532. doi: 10.1590/S2237-96222025v34e20240532.en (PMC12342713; doi:10.1590/S2237-96222025v34e20240532.en)
Supplement: Translation

# Translation: Adequação de um hospital pediátrico às recomendações dos guias alimentares brasileiros, Niterói, 2023

**DOI:** 10.1590/S2237-96222025v34e20240532.pt

**Authors:** Amine Farias Costa, Letícia Ferreira Tavares, Paulo Cesar Pereira de Castro, Raphael Barreto da Conceição Barbosa, Olivia Souza Honório, Ana Carolina Rocha Oliveira, Letícia de Oliveira Cardoso

**Affiliations:** 1 Instituto Nacional de Câncer, Hospital do Câncer II, Rio de Janeiro, RJ, Brasil; 2 Universidade Federal do Rio de Janeiro, Instituto de Nutrição Josué de Castro, Departamento de Gastronomia, Rio de Janeiro, RJ, Brasil; 3 Universidade Federal do Rio de Janeiro, Instituto de Nutrição Josué de Castro, Departamento de Nutrição Social Aplicada, Rio de Janeiro, RJ, Brasil; 4 Instituto Desiderata, Rio de Janeiro, RJ, Brasil; 5 Fundação Oswaldo Cruz, Escola Nacional de Saúde Pública Sergio Arouca, Rio de Janeiro, RJ, Brasil

**Keywords:** Ambiente, Alimento, Hospital, Pediatria, Estudo Observacional

## Abstract

**Objetivo:**

Analisar a adequação de um hospital pediátrico, de um município de grande porte da região metropolitana do Rio de Janeiro, às recomendações dos guias alimentares no que tange ao contrato de prestação de serviço, prescrição dietética e aspectos do ambiente alimentar.

**Métodos:**

Estudo observacional que analisou quatro dimensões do ambiente alimentar hospitalar e do seu entorno: legislação vigente e contrato de prestação de serviços de alimentação; dietas prescritas, cardápio elaborado e fichas técnicas das preparações; infraestrutura da Unidade de Alimentação e Nutrição; e a venda e propaganda de alimentos no ambiente alimentar interno e externo. Foram realizadas visitas técnicas para avaliação documental, análise de cardápios e observação direta, utilizando instrumentos previamente validados.

**Resultados:**

O contrato de prestação de serviços estava totalmente conforme para fórmulas infantis e dietas enterais, e parcialmente conforme (81%) para o cardápio. A maioria das preparações analisadas foi classificada como de alta qualidade ou qualidade intermediária (77%). Na infraestrutura da Unidade de Alimentação e Nutrição, 90%, 89% e 71% das respostas foram positivas para itens imprescindíveis, recomendáveis e necessários, respectivamente. O ambiente alimentar do hospital se destacou pela oferta de alimentos in natura e minimamente processados. Contudo, havia uma grande variedade de alimentos ultraprocessados disponíveis em máquinas de autosserviço e com vendedores ambulantes.

**Conclusão:**

Apesar de um ambiente alimentar satisfatório, identificou-se a presença e comercialização de alimentos ultraprocessados no hospital e seu entorno.

**Aspectos éticos**

Esta pesquisa utilizou bancos de dados de domínio público e anonimizados.:

## Introdução

A alimentação desempenha papel fundamental ao longo de todas as fases da vida, especialmente nos primeiros anos, e influencia diretamente o processo de crescimento e desenvolvimento, a formação de novos hábitos e a manutenção da saúde e a formação de capital humano de uma sociedade. A alimentação inadequada, e consequentemente a má nutrição, em suas diferentes expressões, desde desnutrição à obesidade, é um dos principais problemas da saúde pública atualmente (1-3). 

O Brasil tem investido nas últimas décadas na implementação de políticas públicas de promoção, proteção e apoio à alimentação saudável para o público infanto-juvenil, entretanto, apesar dos avanços, o consumo de alimentos ultraprocessados segue crescendo nessa faixa etária. E esse aumento, está associado, dentre outras razões, à exposição precoce a esses alimentos, em detrimento do consumo de alimentos in natura ou minimamente processados (1,4). O consumo de alimentos ultraprocessados tem sido associado a diversos desfechos em saúde deste público, como excesso de peso, obesidade (4,5), comorbidades cardiometabólicas (6,7).

O ambiente alimentar é caracterizado pelo conjunto de condições físicas, econômicas, políticas e socioculturais que influenciam diretamente as escolhas alimentares e o estado nutricional de indivíduos, inclusive crianças e adolescentes (8,9).

Os hospitais, e seus respectivos locais de alimentação, são considerados ambientes alimentares organizacionais, e são alvo de políticas públicas e recomendações nacionais e internacionais para a promoção de hábitos alimentares adequados e saudáveis (10,11). 

A atual Política Nacional de Alimentação e Nutrição brasileira tem entre suas diretrizes a criação e promoção de ambientes alimentares favoráveis à saúde nos quais indivíduos e comunidades possam exercer escolhas saudáveis, e prevê a implementação de ações que protejam os indivíduos de ambientes que fomentem escolhas e práticas pouco saudáveis, por meio de medidas regulatórias (12,13). 

Considerando que o hospital, como instituição, atua como local de recuperação da saúde dos indivíduos e de incentivo a hábitos de vida saudáveis, e que é fundamental que suas práticas estejam consonâncias com as orientações preconizadas pelo Sistema Único de Saúde (SUS), o objetivo deste estudo foi analisar a adequação de um hospital pediátrico, de um município de grande porte da região metropolitana do Rio de Janeiro, às recomendações dos guias alimentares no que tange ao contrato de prestação de serviço, prescrição dietética e aspectos do ambiente alimentar. 

## Métodos

### Desenho

Trata-se de um estudo observacional, transversal, de base hospitalar, realizado entre os meses de janeiro e maio de 2023.

### Contexto e tamanho do estudo

O estudo foi conduzido em um hospital público pediátrico localizado no município de Niterói, região metropolitana do Estado do Rio de Janeiro, Brasil. O hospital avaliado é referência em atendimentos de pediatria na região, realizando, mensalmente, cerca de 6 mil atendimentos na emergência, 2 mil consultas especializadas e uma média de 180 internações (14). 

A adequação às recomendações dos guias alimentares foi avaliada por meio de visitas técnicas locais, dividida em quatro módulos: 1. avaliação da legislação vigente e do contrato de prestação de serviços de alimentação e nutrição implementado no hospital; 2. avaliação das dietas prescritas, do cardápio elaborado, das fichas técnicas e sua comparação com as diretrizes do contrato; 3. avaliação da infraestrutura da Unidade de Alimentação e Nutrição e do refeitório para confecção e distribuição das refeições; 4. avaliação do ambiente alimentar interno e externo no que tange a venda e propaganda de alimentos. 

### Variáveis

### **Módulo 1**:

Foi realizada uma análise do contrato de prestação de serviços de alimentação e nutrição implementando no hospital e legislações vigentes sobre alimentação no âmbito dos hospitais pediátricos, visando: (a) avaliação das orientações para a elaboração de cardápios infantis, assim como a sua estrutura, composição, frequência de oferta de alimentos, características das dietas hospitalares e itens permitidos e proibidos; (b) avaliação do preparo, composição (essencial e de ingredientes opcionais) e rotulagem de fórmulas infantis; (c) avaliação da prescrição, preparo, transporte, administração, controle clínico e laboratorial, rotulagem e embalagem das dietas enterais, e demais etapas do processo que envolve a terapia nutricional enteral.

A Tabela 1 apresenta os itens que foram analisados na avaliação dos cardápios, fórmulas infantis e dietas enterais. 

**Tabela 1:** Lista de itens analisados na avaliação dos cardápios, fórmulas infantis e dietas enterais distribuídos ao público atendido em um hospital pediátrico da rede pública de Niterói

Item (quantidade absoluta)	Descrição dos itens
**Cardápios** (14)	
Orientações gerais (7)	- Elaboração de cardápios mensais. - Fichas técnicas das preparações alimentícias.^a^ - Previsão de cardápios diferenciados para datas festivas. - Fixação do cardápio em local visível. - Alteração do cardápio com antecedência mínima de 24 horas. - Previsão de molhos e temperos para aumentar a aceitabilidade. - Previsão de gêneros alimentícios especiais para patologias.
Estrutura (1)	- Composição básica de refeições para crianças.
Composição e frequência (4)	- Nível de processamento dos alimentos.^a^ - Grupos alimentares. - Frequência de oferta dos grupos alimentares. - Composição nutricional.
Características das dietas hospitalares (2)	- Dietas básicas. - Dietas de restrição.
Itens alimentares (2)	- Permitidos. - Proibidos.^a^
**Fórmulas Infantis** (16)	
Definição (4)	- Fórmulas infantis para lactentes. - Fórmulas infantis de seguimento para lactentes e crianças de primeira infância. - Fórmulas infantis para lactentes destinadas a necessidades dietoterápicas específicas. - Fórmulas infantis de seguimento para lactentes e crianças de primeira infância destinadas a necessidades dietoterápicas específicas.
Preparo (2)	- Instruções do rótulo. - Quantidade de acordo com peso e idade da criança.
Composição essencial (3)	- Recomendações de macronutrientes. - Recomendações de micronutrientes. - Isenção de glúten.
Ingredientes opcionais (1)	- Regras específicas para utilização de taurina, nucleotídeos, ácido docosahexaenoico, frutooligossacarídeos, galactooligossacarídeos e probióticos.
Rotulagem (5)	- Informação nutricional para 100g ou 100mL. - Ingredientes opcionais com informação nutricional declarada. - Ausência de percentual de valor diário. - Presença de informações obrigatórias nas fórmulas infantis destinadas a necessidades dietoterápicas - Presença de frases de advertência nas fórmulas infantis.
Composto lácteo (1)	- Presença de frases de advertência.
**Dietas Enterais** (45)	
Definição (3)	- Composição definida. - Nutrientes isolados ou combinados; - Sistema aberto ou fechado.
Condições gerais (8)	- Indicação. - Prescrição médica. - Prescrição dietética, preparação. - Conservação e armazenamento. - Transporte. - Administração. - Controle clínico laboratorial. - Avaliação final.
Indicação e prescrição (3)	- Indicação médica. - Prescrição dietoterápica. - Avaliação nutricional prévia.
Preparo e conservação (7)	- Avaliação da prescrição dietética. - Manipulação. - Controle de qualidade. - Conservação. - Certificado de análise para insumos e recipientes. - Rotulagem. - Reserva de amostras.
Transporte e administração (3)	- Manutenção da qualidade. - Transporte. - Administração.
Controle clínico e laboratorial (1)	- Controle clínico e laboratorial do paciente.
Atribuição do nutricionista (1)	- Lista de atribuições privativas.
Boas práticas de preparação (3)	- Preparação da dieta. - Aquisição de insumos e materiais de embalagem. - Aquisição de dietas industrializada.
Especificações para as fórmulas (4)	- Fórmula para nutrição enteral. - Fórmula padrão para nutrição enteral. - Fórmula modificada para nutrição enteral. - Módulo para nutrição enteral.
Rotulagem e embalagem (12)	- Identificação completa do paciente - Composição qualitativa e quantitativa de todos os componentes. - Volume total. - Velocidade de administração. - Via de acesso. - Data e hora da manipulação. - Prazo de validade. - Número sequencial de controle. - Condições de temperatura para conservação. - Nome e número no Conselho Profissional do respectivo responsável técnico pelo processo. - Ausência de informações falsas. - Requisitos de rotulagem específicos para fórmulas modificadas para idades específicas.

^a^Não conforme.

### **Módulo 2**:

Foi realizada uma análise documental para avaliação de todos os gêneros alimentícios e alimentos industrializados adquiridos, além das preparações culinárias elaboradas no local, e distribuídos ao público atendido pelo hospital.

### **Módulo 3**:

As seguintes variáveis relacionadas à infraestrutura da Unidade de Alimentação e Nutrição e refeitório foram avaliadas: documentação e registros; procedimentos operacionais padronizados; instalações físicas; sistema de combate a incêndio; paredes, pisos, portas e janelas; instalações elétricas, iluminação e ventilação; vestiários e sanitários; lavatório para higienização das mãos; lixo, caixas de gordura e esgoto; controle de vetores e pragas urbanas; abastecimento de água; equipamentos e utensílios; higiene do ambiente, das instalações e dos utensílios; higiene dos manipuladores; produção - aquisição e recebimento de matérias-primas; produção - armazenamento; produção - manipulação dos alimentos; produção - distribuição e consumo dos alimentos; controle de qualidade e transporte de alimentos; satisfação dos trabalhadores e usuários.

### **Módulo 4**:

A avaliação do ambiente alimentar do hospital contou com a análise de três tipos de serviços: refeitório, máquinas de autosserviço e comércio ambulante informal presente no entorno imediato do local. 

Características do refeitório como infraestrutura física e de equipamentos, tipo de bebidas, alimentos e preparações ofertados, preços praticados e promoções, propaganda, informação ao consumidor, e horário de funcionamento, foram avaliados. Considerando as máquinas de autosserviço foram avaliadas a oferta, a disponibilidade, a qualidade, a publicidade e o preço de alimentos e bebidas disponíveis. O comércio ambulante no entorno do hospital foi avaliado quanto a sua estrutura, tipos e variedade de alimentos comercializados e publicidade utilizada.

### Fontes de Dados e Mensuração

### **Módulo 1**:

Para a avaliação dos cardápios, fórmulas infantis e dietas enterais foram utilizados o *Guia Alimentar para a População Brasileira* (2) e o *Guia Alimentar para Crianças Brasileiras Menores de 2 Anos* (1), citados a partir daqui como “guias alimentares nacionais”, além de programas e normas do governo brasileiro e legislações de agências regulatórias nacionais (16-24). 

### **Módulo 2**:

Foram analisados os cardápios e preparações de todas as refeições, a composição das mamadeiras elaboradas, as solicitações de compras de gêneros alimentícios, os rótulos dos alimentos industrializados utilizados nas preparações alimentícias ou que foram distribuídos aos pacientes pediátricos do hospital e os mapas de planejamento de refeições da Unidade de Alimentação e Nutrição e dos nutricionistas prescritores. 

Foram analisados três dias aleatórios e não consecutivos (dois dias de semana e um dia de final de semana) para o cardápio do almoço e jantar, para às duas consistências disponíveis (branda e pastosa). A Unidade de Alimentação e Nutrição do hospital não possuía planejamento de cardápio para as pequenas refeições (desjejum, colação, merenda e ceia), portanto, foram analisados os alimentos oferecidos aos pacientes no dia da visita técnica ao hospital. 

### **Módulo 3**:

Os dados foram coletados pela observação e análise da Unidade de Alimentação e Nutrição e refeitório guiadas por um instrumento construído e validado com base nas dimensões de estrutura, processos e resultados, fundamentados na legislação geral sobre alimentos, na literatura científica, e em instrumentos já utilizados por alguns serviços de Vigilância Sanitária (25).

O instrumento utilizado na coleta de dados possui uma lista de 227 itens, dentre os quais 131 são considerados imprescindíveis (aqueles que poderiam influir em grau crítico na qualidade e segurança das refeições e na segurança dos trabalhadores durante o processo de produção), 89 necessários (quando pode influir em grau menos crítico na qualidade e segurança das refeições e na segurança dos trabalhadores durante o processo) e 7 recomendáveis (quando pode influir em grau não crítico na qualidade e na segurança das refeições dos trabalhadores durante o processo).

### **Módulo 4**:

O ambiente alimentar do refeitório foi avaliado utilizando um instrumento elaborado especificamente para a área hospitalar - “Instrumento de Avaliação do Ambiente Alimentar Hospitalar”, que em análise prévia apresentou boa confiabilidade no contexto em que foi aplicado (26). O instrumento, do tipo check-list, contém 8 blocos de avaliação do ambiente alimentar, e para esta análise, somente o bloco D, destinado à avaliação do refeitório hospitalar, foi utilizado. O bloco utilizado contém 41 itens que foram respondidos, baseados na observação do ambiente avaliado. 

Para avaliação das máquinas de autosserviço foi utilizado um instrumento validado, do tipo check-list, que contém 3 blocos: identificação do avaliador, do estabelecimento, do local do estudo e da coleta de dados; informações sobre a máquina de autosserviço, como localização, alimentos e/ou bebidas presentes, marca específica, formas de pagamento e presença de propaganda; e informações sobre os itens alimentares disponíveis (25 opções dentre alimentos e bebidas). (27-28) 

O comércio ambulante no entorno do hospital foi avaliado utilizando um instrumento elaborado para a avaliação do comércio ambulante no entorno de escolas particulares brasileiras (29). O instrumento, do tipo check-list, contém 129 itens, divididos em 3 seções, que compreendem questões sobre dados gerais do comércio ambulante de alimentos; dados sociodemográficos dos comerciantes de rua; estrutura do comércio (carrinho, barraca, e outros); alimentos e bebidas comercializados, com detalhes para a variedade ofertada, tamanho e preço das porções, e estratégias de venda; publicidade utilizada para os alimentos comercializados e se o mesmo já foi impedido ou proibido de atuar no local por parte do poder público, polícia ou pela população.

### Métodos Estatísticos

### **Módulo 1**:

As informações coletadas nos documentos utilizados no hospital foram avaliadas quanto à conformidade (conforme ou não conforme) em relação às normas e legislações utilizados como referência para estas análises, e seu resultado apresentado como frequência absoluta e relativa.

### **Módulo 2**:

O Escore para Avaliação Qualitativa de Preparação (30) foi utilizado para classificar cada item alimentar de acordo com o seu grau de processamento industrial e seguindo as recomendações dos guias alimentares nacionais (1,4). A avaliação das preparações foi baseada no questionário composto por dez questões que avaliaram e pontuaram, além do processamento industrial do ingrediente, o uso de ingredientes in natura como principais e em maior quantidade; a presença ou ausência de proteína animal, ou peixe com menos gordura; o uso de cereais e sementes em sua forma integral; a presença de açúcar, rapadura, mel ou melado; e a presença de frituras por imersão em óleo. Para aplicação desta ferramenta, utilizou-se a planilha do Microsoft Excel disponibilizada pelas autoras do método que organizada as preparações por grupo (salada, prato principal, acompanhamento, guarnição e sobremesa). Os ingredientes de cada preparação e as informações dos rótulos de cada produto utilizado na preparação foram incluídos nesta planilha.

A pontuação e classificação das preparações de acordo com seu escore foi preenchida automaticamente após responder cada pergunta. Cada preparação alimentar foi classificada de acordo com o Escore para Avaliação Qualitativa de Preparação a saber: ≥11, preparação de alta qualidade; 6 a 10, qualidade intermediária; 0 a 5, baixa qualidade; ≤-1, muito baixa qualidade (30).

### **Módulo 3**:

Foi utilizado o percentual de respostas positivas para todos os itens avaliados no hospital. O estabelecimento é classificado em boas condições quando possui 91% ou mais de respostas positivas para os itens imprescindíveis e 71% ou mais para os itens necessários, e é classificado em condições satisfatórias quando possui 70% a 90% de respostas positivas para os itens imprescindíveis e 50% a 70% para os itens necessários (25).

### **Módulo 4**:

Foi utilizado o indicador razão entre a disponibilidade de alimentos ultraprocessados e preparações culinárias contendo estes alimentos e alimentos in natura, minimamente processados ou processados e preparações culinárias baseadas nestes alimentos para avaliação da disponibilidade de alimentos das máquinas de autosserviço e do comércio ambulante (31).

## Resultados

Considerando os documentos e legislações de referência avaliados no módulo 1, o contrato de prestação de serviços de alimentação e nutrição implementado no hospital estava totalmente conforme (Tabela 1) no que se refere às fórmulas infantis e dietas enterais. No que se refere ao cardápio, 16 itens foram avaliados e 3 (19%) foram classificados como não conforme - ausência de fichas técnicas das preparações, utilização de alimentos ultraprocessados, e utilização de itens proibidos (sal e açúcar). Apesar do contrato enfatizar que a dieta do paciente deve seguir as recomendações nutricionais, não há referência aos guias alimentares nacionais.

A maioria das preparações analisadas foi classificada como de alta qualidade ou qualidade intermediária (77%). Todas as preparações salgadas, distribuídas no almoço e jantar, foram classificadas como de alta qualidade (42%) segundo o escore. As preparações classificadas como de qualidade intermediária (35%), baixa qualidade (12%) e muito baixa qualidade (10%) foram aquelas que não possuíam alimentos in natura em sua composição, e aquelas que eram adicionadas de açúcares, adoçantes artificiais ou alimentos ultraprocessados (Tabela 2).

**Tabela 2:** Escore para avaliação qualitativa dos gêneros alimentícios, alimentos industrializados e preparações culinárias elaboradas e distribuídos ao público atendido em um hospital pediátrico da rede pública de Niterói

Preparação	Escore
Alta qualidade	
Bolo de batata com frango	13
Frutas variadas (inteira, creme ou papa)	13
Legumes variados tipo sauté	13
Suco de laranja	13
Purê de legumes variados	13
Arroz branco	12
Carne vermelha (bife ou moída)	12
Feijão mulatinho	12
Frango (filé de peito grelhado ou desfiado)	12
Macarrão	12
Mamadeira: água, leite de vaca integral em pó (13%)	12
Doce de banana	11
Mamadeira: leite de vaca integral, aveia (5%), açúcar (5%)	11
Mingau de aveia (leite de vaca integral, aveia e açúcar)	11
Suco de fruta (polpa de fruta congelada, água e açúcar)	11
Omelete de legumes	12
Peixe grelhado ou desfiado	12
Qualidade intermediária	
Doce manjar	10
Leite integral com café e açúcar	10
Mamadeira: leite de vaca integral, amido de milho (5%), açúcar (5%)	10
Mingau de maisena (leite de vaca integral, amido de milho e açúcar)	10
Vitamina de fruta (leite de vaca integral, fruta e açúcar)	10
Suco de fruta (polpa de fruta congelada, água e adoçante artificial)	7
Leite integral com café e adoçante artificial	6
Leite integral com achocolatado normal ou diet	6
Mamadeira: leite de vaca integral, farinha láctea^a^ (5%)	6
Mamadeira: leite de vaca integral (75%), água, farinha multicereais^a^ (5%), maltodextrina (5%)	6
Mingau de aveia (leite de vaca integral, aveia e adoçante artificial)	6
Mingau de farinha láctea (leite de vaca integral e farinha láctea^a^)	6
Mingau de maisena (leite de vaca integral, amido de milho e adoçante)	6
Vitamina de fruta (leite de vaca integral, fruta e adoçante artificial)	6
Baixa qualidade	
Mamadeira: leite de vaca integral, farinha multicereais^a^ (5%), açúcar (5%)	4
Mamadeira: leite de vaca integral, farinha de arroz^a^ (5%), açúcar (5%)	4
Mamadeira: leite de vaca integral, farinha multicereais^a^ (5%), açúcar (5%)	4
Mamadeira: leite de vaca integral, farinha de arroz^a^ (5%), açúcar (5%)	4
Pão (francês ou careca) com queijo minas	1
Muito baixa qualidade	
Pão (francês ou careca) com margarina	-5
Pão (francês ou careca) com queijo prato	-5
Biscoito salgado ou doce	-6
Doce “mini-saia” (pudim em pó e gelatina em pó)^a^	-7

^a^Produtos ultraprocessados em pó.

Com relação ao módulo 2, as preparações não possuíam fichas técnicas, portanto, as quantidades adicionadas de cada ingrediente variavam de acordo com o funcionário que elaborava a preparação. Contudo, a quantidade dos ingredientes não faz parte do cálculo do Escore para Avaliação Qualitativa de Preparação, e somente a informação da presença ou ausência de determinado ingrediente.

Das 227 questões avaliadas no módulo 3, o percentual de respostas positivas para os itens imprescindíveis, recomendáveis e necessários foi de 90%, 89% e 71%, respectivamente, apresentando, assim, condições satisfatórias no atendimento dos itens imprescindíveis, e boas condições no atendimento dos itens necessários. 

As inconformidades encontradas estão concentradas na ausência de registros de temperatura, calibração de equipamentos e análises microbiológicas; presença de vetores; e inadequações em áreas físicas (Tabela 3).

**Tabela 3:** Avaliação da infraestrutura da Unidade de Alimentação e Nutrição e refeitório hospitalar de um hospital pediátrico da rede pública de Niterói: itens com respostas negativas e classificação correspondente

Classificação do item^a^ (frequência relativa)	Itens com resposta negativa
Imprescindível (5%)	- Local livre de pragas urbanas. - Carros térmicos de distribuição mantêm a temperatura de no mínimo 65ºC. - Verificadas e registradas as temperaturas das matérias-primas perecíveis no ato do recebimento. - Armazenamento permite a ventilação, limpeza e quando for o caso higienização do local. - Eficácia do tratamento térmico avaliada pela verificação da temperatura e do tempo utilizados e, quando aplicável, pelas mudanças na textura e cor na parte central do alimento. - Alimentos em reaquecimento atingem 74ºC em seu interior ou são submetidos a faixa de tempo x temperatura que garanta sua segurança. - No caso de resultados das análises serem insatisfatórias, quais as medidas tomadas.
Necessário (10%)	- Possui Projeto arquitetônico aprovado pela Vigilância Sanitária. - Possui Programa de manutenção e calibração de equipamentos. - Registro de manutenção e calibração de equipamentos. - Procedimentos Operacionais Padronizados para calibração de equipamentos. - Vestiários e Sanitários respeitam as exigências de instalações gerais. - Vestiários dotados de armários que não apresentaram bom estado de conservação. - As luvas térmicas são usadas de forma a evitar o contato direto com os alimentos. - No armazenamento à temperatura ambiente a capacidade física dos depósitos é suficiente. - Elevadores para transporte da refeição (quando for o caso) são de uso exclusivo.
Recomendável (29%)	- O estabelecimento mede e avalia a satisfação dos trabalhadores. - Existem incentivos à formação dos profissionais oferecidos pelo estabelecimento aos funcionários.

^a^Foram avaliados 227 itens, entre janeiro e maio de 2023, sendo: 131 itens classificados como imprescindíveis, 89 necessários e 7 recomendáveis. Os itens imprescindíveis podem influir em grau crítico na qualidade e segurança das refeições e na segurança dos trabalhadores durante o processo de produção; os itens necessários podem influir em grau menos crítico na qualidade e segurança das refeições e na segurança dos trabalhadores durante o processo; e o itens recomendáveis podem influir em grau não crítico na qualidade e na segurança das refeições dos trabalhadores durante o processo.

Foram encontrados um refeitório e duas máquinas de autosserviço nas avaliações do módulo 4. No entorno, estavam presentes dois vendedores ambulantes de alimentos e bebidas.

O refeitório possui climatização e mobiliário para a realização das refeições no local, o cardápio diário é divulgado aos usuários e é composto por 4 refeições, com a oferta de hortaliças, cereais e leguminosas nas grandes refeições, e de café e pães nas pequenas refeições. Não foi identificada a oferta de frutas in natura no cardápio avaliado. 

No entanto, as máquinas de autosserviço são os principais pontos de distribuição de alimentos e bebidas disponíveis para livre acesso a todos presentes no hospital, com a oferta de alimentos ultraprocessados predominante, e ainda com incentivos publicitários ao consumo dos mesmos. Dentre os alimentos ultraprocessados disponíveis para aquisição foram encontradas bebidas ultraprocessadas prontas para o consumo, chocolates, balas, e outras guloseimas, biscoitos recheados e salgadinhos de pacote. O mesmo perfil de disponibilidade de alimentos foi encontrado no comércio ambulante. 

Os resultados do indicador razão entre a disponibilidade de alimentos ultraprocessados e preparações culinárias contendo estes alimentos e alimentos in natura, minimamente processados ou processados e preparações culinárias baseadas nestes alimentos evidenciam que há predominância de disponibilidade de alimentos ultraporcessados nas máquinas de autosserviço e nos ambulantes. Os ambulantes disponibilizam de 1,3 a 3,5 vezes mais alimentos ultraprocessados e preparações culinárias contendo estes alimentos do que alimentos in natura, minimamente processados ou processados e preparações culinárias baseadas nestes alimentos. Uma máquina de autosserviço disponibiliza 10 alimentos ultraporcessados e nenhum alimento in natura, minimamente processados ou processados e preparações culinárias baseadas nestes alimentos e a outra disponibiliza 50% mais alimentos ultraporcessados. 

## Discussão

Este estudo apresentou dados sobre a avaliação do ambiente alimentar de um hospital pediátrico localizado na região metropolitana do Rio de Janeiro. Os resultados evidenciam a aquisição, distribuição, publicidade e venda de alimentos ultraprocessados para o público infanto-juvenil atendidos no local, assim como para seus responsáveis e trabalhadores do local. Destaca-se também que o contrato de prestação de serviços do hospital orienta seguir as recomendações nutricionais, mas não referência os guias alimentares nacionais (1,2).

Ao analisar as diretrizes do contrato vigente e os instrumentos gerenciais utilizados, nota-se adequação, em sua maioria, às legislações e documentos nacionais da área de alimentação e nutrição. Porém, visualiza-se a oferta e distribuição de alimentos ultraprocessados para os usuários, e ao observar a análise do ambiente alimentar, nota-se também a publicidade e venda de alimentos ultraprocessados no interior e no entorno do hospital. Dessa forma, tanto a oferta quanto os alimentos disponibilizados no ambiente vão contra as recomendações dos guias alimentares que preconizam a oferta de alimentos in natura e minimamente processados e orientam evitar os alimentos ultraprocessados. 

O consumo de alimentos ultraprocessados está relacionado com a incidência de obesidade e doenças crônicas não transmissíveis em adultos e no público infanto-juvenil (2). Assim, considerando que o ambiente hospitalar tem como finalidade, dentre outras, a promoção, prevenção, recuperação e proteção à saúde, a oferta, venda e publicidade de alimentos ultraprocessados neste ambiente, mesmo havendo um conjunto de evidências que destacam seus malefícios à saúde, afasta o local dos seus objetivos.

Uma revisão narrativa, a partir de estudos realizados nos EUA e no Reino Unido, apontou para uma diversidade de alimentos disponíveis nos comércios dentro dos hospitais (restaurantes, lanchonetes e máquinas de autosserviços), mas com uma baixa disponibilidade de alimentos saudáveis. O estudo aponta ainda que medidas regulatórias têm um efeito positivo tanto no aumento da aquisição de alimentos saudáveis quanto na redução de alimentos não saudáveis (32).

No Brasil, um estudo que objetivou caracterizar o ambiente alimentar da rede de hospitais públicos, geridos pela prefeitura da cidade do Rio de Janeiro (n=24), concluiu que o ambiente alimentar dos hospitais estudados não são promotores de hábitos alimentares saudáveis. Foi encontrada uma alta disponibilidade de alimentos ultraprocessados no ambiente hospitalar, e que a publicidade desses alimentos é frequente, estimulando o consumo de alimentos não saudáveis (33). Os resultados dialogam com os aqui apresentados ao observarmos os alimentos comercializados no interior do hospital.

A iniciativa INFORMAS (International Network for Food and Obesity/non-communicable disease Research, Monitoring and Action Support) defende que os ambientes organizados e geridos pelo poder público deveriam ser regidos por políticas alimentares que regulem o que deve ou não ser ofertado. Destaca ainda que a qualidade nutricional dos alimentos disponibilizados e/ou comercializados em espaços públicos deveriam estar em consonância com as diretrizes, normas ou orientações nutricionais estabelecidas pelo país (34).

Ressalta-se ainda, que medidas como a proibição e restrição da oferta, comercialização e publicidade de alimentos ultraprocessados têm sido recomendadas por diferentes organismos internacionais no que tange a contenção da obesidade, especialmente do público infantil. Nos últimos anos, organizações de governança internacional têm recomendado a proteção dos ambientes onde crianças e adolescentes estão inseridos, e no contexto dos ambientes alimentares destacam a necessidade da construção de ambientes alimentares saudáveis, especialmente os ambientes organizacionais (35-37). 

 No Brasil, algumas ações de regulamentação da disponibilidade, acesso, distribuição, propaganda e publicidade de alimentos ultraprocessados, baseadas em evidências científicas e nos guias alimentares nacionais (1,2), foram propostas nos últimos anos e poderiam ser adotadas no sentido de estabelecer uma consonância entre o que é preconizado pelo Ministério da Saúde e o que é ofertado nos estabelecimentos de saúde que fazem parte do SUS. O Ministério da Saúde instituiu diretrizes de promoção da alimentação adequada, saudável e sustentável a serem adotadas nas ações de promoção da saúde e qualidade de vida no trabalho e em ambientes que comercializam, produzem ou ofertam alimentação no âmbito do Ministério (38), não regulando também o que deveria ser disponibilizado nos estabelecimentos da rede do SUS. Para o público infanto-juvenil, o Programa Nacional de Alimentação Escolar prevê a proibição, na alimentação escolar, da oferta de alimentos ultraprocessados e da adição de açúcares e adoçantes artificiais nas preparações culinárias e bebidas para as crianças de até três anos de idade (39), mostrando assim a viabilidade e importância de medidas regulatórias dentro de sistemas da rede pública. 

Até o presente momento, não há estudos que avaliem o ambiente alimentar de hospitais pediátricos. O ineditismo do assunto requer discussão contínua e a necessidade de cooperação entre diversos atores. Nesse sentido, os resultados do presente estudo mobilizaram os pesquisadores envolvidos na equipe de pesquisa e um painel de juízes composto por especialistas de diferentes áreas, que extrapolam o campo da saúde e a esfera governamental, na construção de uma Nota Técnica, baseada nos guias alimentares nacionais, que pretende orientar a regulamentação do ambiente alimentar hospitalar (40).

O presente estudo também apresenta limitações. Trata-se de um estudo seccional em uma unidade hospitalar, que está suscetível aos possíveis vieses de observação. Também é preciso destacar que a avaliação do ambiente alimentar no entorno pode sofrer uma alteração, dado que foi analisado o comércio ambulante informal na calçada do estabelecimento de saúde, e que a presença do mesmo é suscetível a fatores como condições de tempo e horário. No entanto, para minimizar os riscos desses vieses foram utilizados instrumentos validados e padronizados, protocolos de pesquisa e os pesquisadores de campo foram treinados, bem como a coleta de ambiente alimentar aconteceu em um dia sem chuva, em horário comercial. 

A avaliação realizada demonstrou que o ambiente alimentar do hospital, em geral, foi satisfatório, no que tange ao cumprimento das legislações, infraestrutura, qualidade dos serviços prestados e alimentação fornecida, porém, com a oferta, venda e publicidade de alimentos ultraprocessados no interior e no entorno do local. Espera-se que esse estudo contribua para subsidiar o aperfeiçoamento de medidas regulatórias em ambientes alimentares institucionais, visando o acesso a uma alimentação de boa qualidade, saudável e sustentável a todos os usuários desses locais.

## Data Availability

O estudo apresentado não dispõe de um banco de dados. Informações adicionais estão disponíveis mediante solicitação aos autores.
